# *Aphaenogasterillyrica*, a new species from the mountains of the Balkan Peninsula (Hymenoptera, Formicidae)

**DOI:** 10.3897/zookeys.862.32946

**Published:** 2019-06-09

**Authors:** Gregor Bračko, Albena Lapeva-Gjonova, Sebastian Salata, Lech Borowiec, Slavko Polak

**Affiliations:** 1 University of Ljubljana, Biotechnical Faculty, Department of Biology, Jamnikarjeva 101, SI-1000 Ljubljana, Slovenia University of Ljubljana Ljubljana Slovenia; 2 Department of Zoology and Anthropology, Faculty of Biology, Sofia University, 8 Dragan Tsankov Blvd., 1164 Sofia, Bulgaria Sofia University Sofia Bulgaria; 3 Institute for Agricultural and Forest Environment, Polish Academy of Sciences, Bukowska 19, 60-809 Poznań, Poland Institute for Agricultural and Forest Environment, Polish Academy of Sciences Poznań Poland; 4 Department of Biodiversity and Evolutionary Taxonomy, University of Wrocław, Przybyszewskiego 65, 51-148 Wrocław, Poland University of Wrocław Wrocław Poland; 5 Notranjska Museum Postojna, Kolodvorska cesta 3, 6230 Postojna, Slovenia Notranjska Museum Postojna Postojna Slovenia

**Keywords:** *Aphaenogastersubterranea* species group, Mediterranean Region, Superficial Subterranean Habitat, taxonomy

## Abstract

*Aphaenogasterillyrica***sp. nov.**, a member of the *A.subterranea* species group, is described from Dinaric Alps of Slovenia and Croatia, from Golešnica Mt. in north Macedonia, Osogovo-Belasica Massif of southwestern Bulgaria, and from Kerkini Mts. of Greek Macedonia. It is characterised by large body size, moderately sculptured head, elevated mesonotum, and long propodeal spines. Its habitat preferences are discussed. A key to the *Aphaenogastergraeca* complex is provided.

## Introduction

*Aphaenogaster* Mayr, 1853 is a worldwide genus, which includes 226 species and subspecies. Among them, 128 species and subspecies are known from the Palearctic Region (Bolton 2018) and 43 from Europe (Borowiec 2014, Borowiec and Salata 2014, 2018, Salata and Borowiec 2016, Borowiec et al. 2019, Gómez et al. 2018). Schulz (1994) proposed the division of some west Palearctic taxa of *Aphaenogaster* into species groups. Since then, several taxonomists introduced new species groups or redefined those proposed by Schulz (1994) (e.g., Kiran et al. 2008, Boer 2013, Borowiec and Salata 2014, Salata and Borowiec 2018, Alicata and Schifani 2019). One of the most characteristic and easily distinguished group is formed by taxa defined by Schulz (1994) as the *Aphaenogastersubterranea* species group. Twelve species were included, and recently one more taxon (*Aphaenogastersubterraneafiorii* Emery) was transferred to this group by Salata and Borowiec (2018). However, most recently Alicata and Schifani (2019) distinguished a new species group comprising taxa formerly assigned to the *A.subterranea* group. In general, most of species belonging to the *A.subterranea* group are characterised by yellowish red to dark brown body colouration, mostly medium-sized body length, weakly sculptured and partly smooth head and mesosoma, with head dorsal surface being from completely sculptured and matt to partly smooth and shiny, scape reaching over the occipital margin of head, middle funicular segments at most 1.5 times as long as wide, and head rectangularly rounded in frontal view.

The material from the broader Mediterranean region that was recently studied showed that this group is more speciose and comprises several morphologically cryptic or subcryptic species, especially from the complex of species close to *A.subterranea* (Latreille, 1798), which is now under detailed study (Borowiec, Csősz, Galkowski & Salata unpublished data). One of the morphospecies of the newly studied material, collected from the mountains of the Balkan Peninsula in Bulgaria, Croatia, Greece, North Macedonia, and Slovenia, differs from all known European taxa of the *A.subterranea* group. Its separation, based on strong morphological features, is possible, and we present its description below.

## Materials and methods

Ant material of the new species, for which we have more detailed data, was sampled either by hand sampling (Bulgarian sample from 2013 and Slovenian sample from 2018) or by applying surface pitfall traps (Bulgarian samples from 2002, 2009, and 2014) or subterranean pitfall traps sensu Wong and Guénard (2017) (Slovenian sample from 2003). In the latter, pitfall traps were glass jars with a saturated salt solution as a fixative, baited with rotting meat and fermented cheese in a vial, as described by Giachino and Vailati (2010). We set them in the so-called Superficial Subterranean Habitat (Juberthie et al. 1980; 1981) among the limestone rocks and soil at depths of 30–50 cm and left them for approximately half a year (from autumn 2002 to May 2003).

A total of 48 specimens was examined. Additionally, we examined 31 specimens of the most closely related species *A.graeca* Schulz, endemic to the Olympus Massif and adjacent mountain ranges Pieria and Kato Olympus. Specimens were compared using standard methods of comparative morphology. For measurement purposes we randomly chose ten specimens, which represented the geographical and morphological variation of the species. The same method was applied to the specimens of *A.graeca*. Photographs were taken using a Nikon SMZ 1500 stereomicroscope, Nikon D5200 photo camera, and Helicon Focus software. All provided label data for the holotype are in original spelling; a vertical bar (|) separates data on different rows and double vertical bars (||) separate labels. All locality points that did not have latitude-longitude information with a paratype were georeferenced using online mapping resources.

**Repository abbreviations**:

**BFUS** Biological Faculty, University of Sofia, Bulgaria;

**DBET** Department of Biodiversity and Evolutionary Taxonomy, University of Wrocław, Poland;

**BFUL** Biotechnical Faculty, Department of Biology, University of Ljubljana, Slovenia;

**MNHW** Museum of Natural History, University of Wrocław, Wrocław, Poland;

**PW** coll. P. Werner, Prague, Czech Republic.

**Measurements: all measurements are given in mm.**

**EL** eye length; measured along the maximum vertical diameter of eye;

**EW** eye width; measured along the maximum horizontal diameter of eye;

**HL** head length; measured in straight line from mid-point of anterior clypeal margin to mid-point of posterior cephalic margin in full-face view;

**HS** arithmetic mean of HL and HW;

**HW** head width; measured in full-face view directly above the eyes;

**ML** mesosoma length; measured as diagonal length from the anterior end of the neck shield to the posterior margin of the propodeal lobe;

**PEH** petiole height; the chord of ventral petiolar profile at node level is the reference line perpendicular to which the maximum height of petiole is measured;

**PEL** petiole length; length of the petiolar node, measured in lateral view from petiolar spiracle to dorso-caudal corner of caudal cylinder;

**PEW** petiole width; maximum width of petiole in dorsal view;

**PNW** pronotum width; maximum width of pronotum in dorsal view;

**PPH** postpetiole height; maximum height of postpetiole in lateral view measured perpendicularly to a line defined by the linear section of the segment border between dorsal and ventral petiolar sclerite;

**PPL** postpetiole length; maximum length of postpetiole in lateral view;

**PPW** postpetiole width; maximum width of postpetiole in dorsal view;

**PSL** propodeal spine length; measured from the centre of the propodeal spiracle to the tip of the propodeal spine in lateral view;

**SDL** spiracle to declivity length; minimum distance from the centre of the propodeal spiracle to the propodeal declivity;

**SL** scape length; maximum straight-line length of scape excluding the articular condyle.

**Indices**:

**HI** (head index). HW/HL × 100.

**SI1** (scape index 1). SL/HL × 100.

**SI2** (scape index 2). SL/HW × 100.

**MI** (mesosoma index). ML/PNW × 100.

**EI** (eye index). (EW+EL)/(HW+HL) × 100.

**PEI** (petiole index). PEL/PEH × 100.

**PPI** (postpetiole index). PPL/PPH × 100.

**PSI** (propodeal spine index). PSL/SDL × 100.

We list the Mediterranean species considered to be members of the *Aphaenogastersubterranea* species group, as defined by Schulz (1994) and Boer (2013), below. However, *A.crocea* André, *A.fiorii* Emery, *A.faureli* Cagniant, *A.hesperia* Santschi, *A sicula* Emery, and the recently described *A.trinacriae* Alicata & Schifani are excluded from the list as they are considered members of a closely related, but distinct group (Alicata and Schifani 2019).

*Aphaenogastergraeca* Schulz, 1994, endemic to Greece

[Holotype (CASENT0911129) and paratypes (CASENT0917360, FOCOL0516, FOCOL1838, FOCOL1839, FOCOL1840) images examined, AntWeb, photos by Will Ericson, Kate Martynova, Christiana Klingenberg available on AntWeb.org].

*Aphaenogasterholtzi* (Emery, 1898), eastern Turkey

[Syntype worker images examined, AntWeb, CASENT0904178, photos by Will Ericson, available on AntWeb.org].

*Aphaenogasterillyrica* sp. nov.

*Aphaenogasterlesbica* Forel, 1913, endemic to Greece

[Syntype worker examined].

*Aphaenogastermaculifrons* Kiran & Aktaç, 2008, western Turkey

[Paratype worker examined].

*Aphaenogastersubterranea* (Latreille, 1798), described from France, recorded from almost the whole western Palearctic Region, probably a complex of cryptic species

[Topotype workers examined, the same locality and series from which neotype has been designated].

*Aphaenogastersubterraneaichnusa* Santschi, 1925, France, Italy, and Spain

[Syntype worker images examined, AntWeb, CASENT0913132, photos by Zach Lieberman, available on AntWeb.org].

## Taxonomy

### Aphaenogaster illyrica sp. nov.

Taxon classificationAnimaliaHymenopteraFormicidae

http://zoobank.org/52996364-A884-45B3-BB10-8C3FC41BDEA6

Figures 1–2 , 3, 4 , 5, 6 , 7–8

#### Material examined.

**Holotype worker**: **SLOVENIA**: Mt. Velika Milanja | MSS | Volovja reber | Ilirska Bistrica, SLO | 45.593N, 14.313E, 1060 m | 23.05.2003, leg. S. Polak (MNHW, holotype no. CASENT0872099).

**Paratypes**: **BULGARIA**: 5 workers (CASENT0872100-CASENT0872104): Maleshevska Mt., Strumyani distr., Dobri Laki vill., 41.58484N, 22.98138E, 650 m, soil traps, along Lebnitsa river, beech and alder trees, 30.07.-20.08.2002, leg. S. Lazarov, T. Ljubomirov (BFUS); 1 worker (CASENT0872105): Belasitsa Mt., Petrch district, Belasitsa hut, 41.370N, 23.187E, 690 m, beech forest, 28.03.2009, leg. R. Bekchiev (BFUS); 15 workers (CASENT0872106-CASENT0872120): Belasitsa Mt., Petrch district, Kamena vill., 41.360N, 23.074E, 500 m, beech forest, along Kamenishka river, soil traps, June 2009, leg. R. Kostova; 02.05.2013, direct sampling, leg. A. Lapeva-Gjonova (BFUS, DBET); 6 workers (CASENT0872121-CASENT0872126): Slavyanka Mt., Sandanski district, Goleshovo vill., 41.42139N, 23.625N, 1094 m, 16.08.2014, leg. A. Lapeva-Gjonova (BFUS); **CROATIA**: 9 workers (CASENT0872127-CASENT0872135): Oltari, Mt. Senjsko bilo, 7 km NW of Krasno, 44.84604N, 15.00298E, 02.06.1992, leg. A. Schulz, K. Vock (DBET, PW); **GREECE**: 3 workers (CASENT0872136- CASENT0872138): [Macedonia] Kerkini Mts., Ano Poroia, 41.28563N, 23.03598E, 28.5.1984, V. Vohralik lgt. (PW, DBET); **NORTH MACEDONIA**: 4 workers (CASENT0872139-CASENT0872142): Golešnica Mts., 2 km S of Aldinci, 41.80189N, 21.42848E, 9.7.2010, 1420 m, V. Vohralik lgt. (DBET, PW); **SLOVENIA**: 1 worker (CASENT0872143): Mt. Velika Milanja, MSS, Volovja reber, Ilirska Bistrica, SLO, 45.593N, 14.313E, 1060 m, 23.05.2003, leg. S. Polak (DBET); 2 workers (CASENT0872144-CASENT0872145): Mt. Velika Milanja, MSS, Volovja reber, Ilirska Bistrica, SLO, 45.593N, 14.313E, 1060 m, 05.10.2018, leg. G. Bračko (BFUL).

#### Differential diagnosis.

The sculpture of head and mesosoma, head shape, scape length, and length of funicular segments place this species into the *Aphaenogastersubterranea* species group. *Aphaenogasterillyrica* differs from other members of this group in the combination of the following features: mesonotum clearly raised above the surface of pronotum, long and thin propodeal spines, as long as or longer than 0.7 length of the first segment of funiculus, elongated mesosoma, large body size (ML more than 1.64 mm, HW more than 1.02 mm), anterolateral sides of pronotum regularly convex, without setose angulations or tubercles, and yellowish brown to rusty brown body colour. In most of the other members of the group (i.e., *A.lesbica* Forel, 1913 from Lesbos, *A.maculifrons* Kiran & Aktaç, 2008 from the western Turkey, *A.subterranea* (Latreille, 1798)), pronotum and mesonotum form a regular convexity, without mesonotum raised above the surface of pronotum, propodeal spines are shorter, not longer than half length of the first segment of antennal funiculus, ML is less than 1.60 mm, and HW less than 1.0 mm.

*Aphaenogasterillyrica* most closely resembles *A.graeca* Schulz, 1994 from Mount Olympus (see Table 1) in morphometric data and general body shape. The new species differs form *A.graeca* in having a brighter and more uniform body colouration (yellowish brown to rusty brown vs. dark brown), weaker head sculpture, which fades laterad, less distinctly sculptured pronotum especially at sides, propodeum smooth and lacking longitudinal rugae on almost of whole lateral surface, and absence of long rugae at the base of the first gaster tergite (Figs 9–12).

**Table 1.** Measurements and indices of *Aphaenogasterillyrica* and *A.graeca*. Values are given as arithmetic mean ± standard deviation (minimum-maximum); n = number of workers; all measurements in mm.

Measurements and indices	*Aphaenogasterillyrica* n = 10	*Aphaenogastergraeca* n = 10
HL	1.319 ± 0.06 (1.218–1.432)	1.373 ± 0.1 (1. 18–1.48)
HW	1.132 ± 0.07 (1.021–1.23)	1.156 ± 0.1 (0.93–1.27)
HS	1.226 ± 0.07 (1.119–1.331)	1.265 ± 0.1 (1.055–1.375)
SL	1.337 ± 0.07 (1.235–1.448)	1.367 ± 0.07 (1.24–1.46)
EL	0.218 ± 0.01 (0.197–0.247)	0.217 ± 0.02 (0.18–0.24)
EW	0.167 ± 0.009 (0.152–0.181)	0.148 ± 0.02 (0.11–0.17)
ML	1.758 ± 0.07 (1.646–1.909)	1.882 ± 0.1 (1.63–2.0)
PSL	0.313 ± 0.03 (0.263–0.378)	0.333 ± 0.05 (0.26–0.42)
SDL	0.203 ± 0.01 (0.181–0.23)	0.224 ± 0.04 (0.18–0.31)
PEL	0.551 ± 0.03 (0.51–0.609)	0.6 ± 0.07 (0.48–0.67)
PPL	0.378 ± 0.02 (0.346–0.395)	0.342 ± 0.03 (0.28–0.37)
PEH	0.36 ± 0.02 (0.329–0.395)	0.38 ± 0.03 (0.33–0.41)
PPH	0.358 ± 0.02 (0.329–0.378)	0.356 ± 0.03 (0.3–0.4)
PNW	0.722 ± 0.04 (0.658–0.79)	0.757 ± 0.07 (0.64–0.88)
PEW	0.266 ± 0.01 (0.246–0.283)	0.273 ± 0.03 (0.22–0.3)
PPW	0.337 ± 0.02 (0.296–0.366)	0.328 ± 0.04 (0.22–0.36)
HI	85.8 ± 2.2 (82.2–89.4)	84.1 ± 2.7 (78.8–86.9)
SI1	101.3 ± 2.9 (96.2–106.7)	99.8 ± 3.0 (95.9–105.1)
SI2	118.1 ± 6.0 (109.2–127.4)	119.0 ± 7.9 (111.8–133.3)
MI	243.6 ± 8.5 (227.1–255.0)	249.3 ± 9.4 (227.3–257.6)
EI	15.7 ± 0.7 (14.6–16.9)	14.4 ± 1.3 (12.3–16.7)
PEI	154.0 ± 5.0 (145.5–161.9)	157.3 ± 8.7 (145.5–169.2)
PPI	105.1 ± 8.1 (93.8–114.3)	96.5 ± 5.3 (89.7–103.2)
PSI	156.4 ± 6.4 (150.0–166.7)	148.9 ± 6.8 (135.4–159.1)

Stout members of the *A.splendida* species group, i.e., *A.festae* Emery, 1915 and its relatives with the mesonotum raised clearly above the surface of pronotum, clearly differ in the yellowish body, short propodeal spines directed distinctly upwards, and elongate segments 2–4 of antennal funiculus, always 1.5 times or more longer than wide.

We also recognise several yet undescribed members of the *A.subterranea* group, which will be a subject for further, more advanced studies. *Aphaenogasterillyrica* is most similar to an undescribed species collected on the island of Cephalonia, especially in its long propodeal spines and mesonotum slightly raised above the surface of pronotum, but the undescribed form differs in having a distinctly microreticulated and dull dorsal and occipital parts of the head surface and dorsum of pronotum, as well as in the anterolateral corners of pronotum bearing setose tubercles.

#### Description of worker.

Measurements: see Table 1.

**Figures 1, 2.**
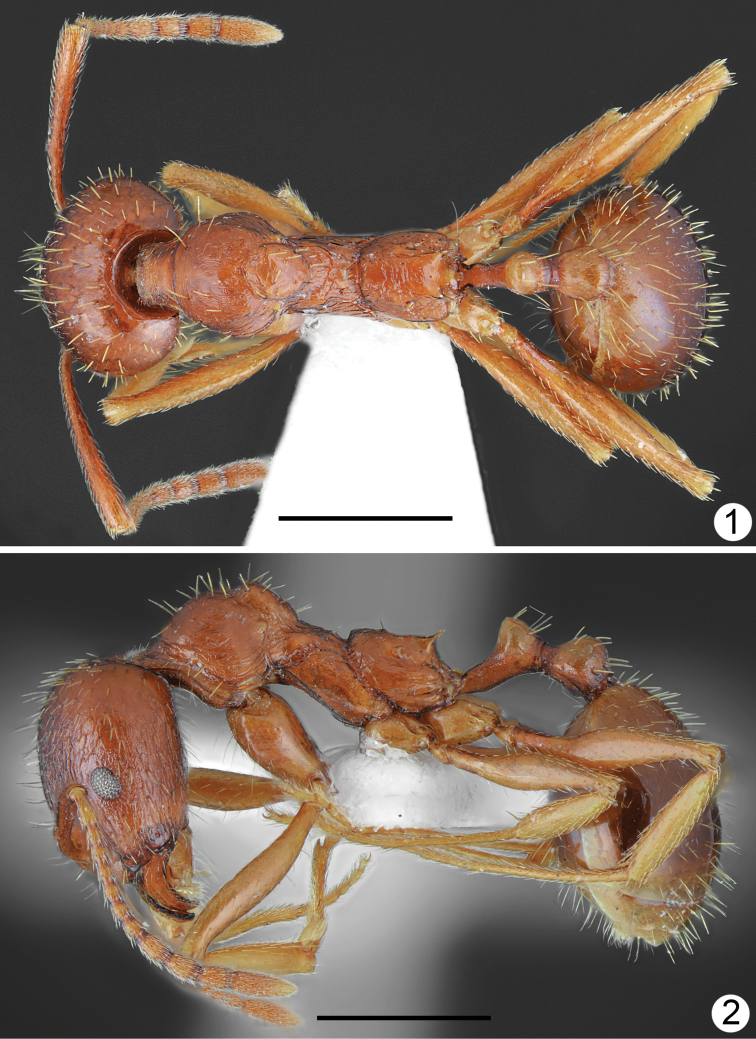
*Aphaenogasterillyrica*, holotype **1** dorsal **2** lateral. Scale bars: 1 mm.

**Figures 3, 4.**
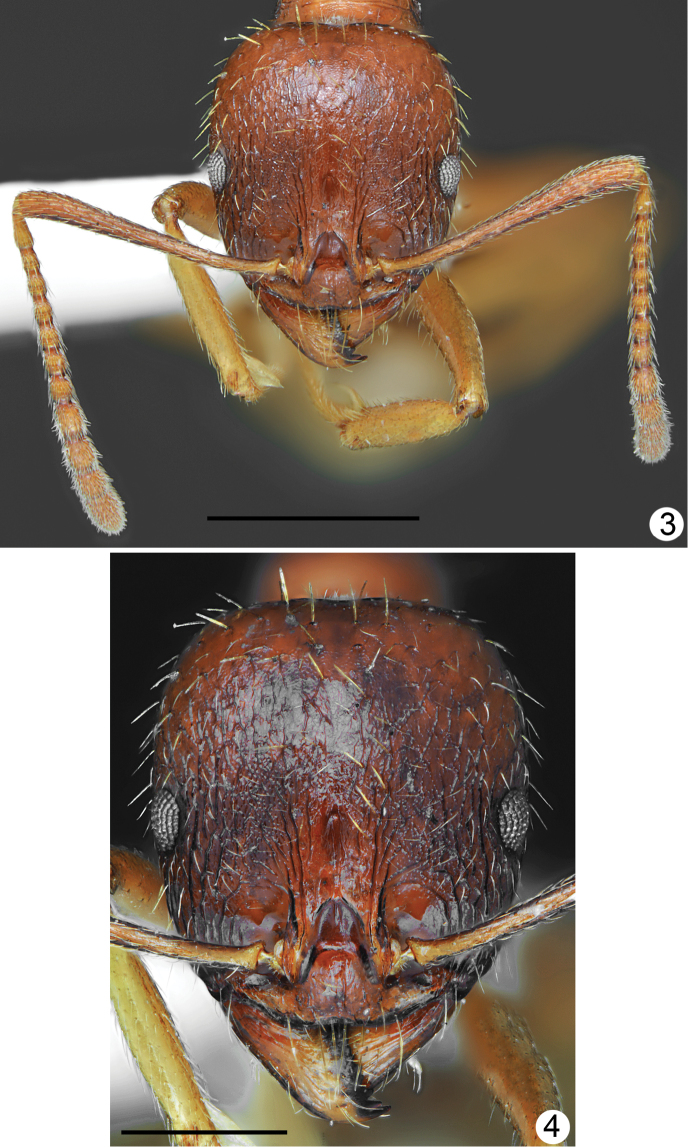
*Aphaenogasterillyrica*, holotype **3** head and antennae **4** head sculpture. Scale bars: 1 mm (**3**), 0.5 mm (**4**).

***Body colouration*.** Head, mesosoma, petiole and postpetiole yellowish brown to rusty brown, frons and area lateral of frontal carinae darker brown. Gaster from yellowish to mostly brown, first tergite yellowish anteriorly and yellowish brown posteriorly, but without distinct border between paler and darker parts, or completely brown. Mandibles yellowish-brown, legs yellow, antennal scapes ochraceous brown with yellowish apex, funiculus ochraceous-yellow (Figs 1, 2). ***Head*.** Approximately 1.2 times as long as wide, lateral margins in frontal view almost parallel behind eyes and evenly rounded at the posterior cephalic corners, posterior margin straight (Fig. 3). Anterior margin of clypeus shallowly emarginated. Eyes small, approximately 0.16 times as long as lateral margin of head, placed in the middle of lateral margin of head (Fig. 4). Scape approximately 1.2 times as long as head width, at base twice narrower than at apex, then gradually widened, without preapical constriction. Funiculus approximately 1.4 times as long as scape, first segment elongated, 2.6 times as long as wide at apex, 0.9 times as long as two subsequent segments combined, segments 2–6 short, 1.2–1.4 times as long as wide, segments 8–10 approximately 1.6 times as long as wide, last 4 segments forming an indistinct club, as long as basal funicular segments 1–7 combined. Mandibles elongate, with distinct striation and with some elongate punctures but shiny, masticatory margin with 7–9 teeth. Clypeus in the middle microreticulated, with short and thin median keel and few indistinct, longitudinal rugae, laterally with distinct longitudinal rugae. Frontal carinae moderately elongate, not reaching half-length of head, subparallel, frontal triangle with median keel and smooth laterally. Frons along the middle with single elevated keel, on sides with 2–3 longitudinal rugae, interspaces microreticulated, moderately shiny. Antennal cavities margined by regular, circular rugae. Central part of head dorsum between eyes with mostly sparse, partly longitudinal and partly irregular rugae, extending to 2/3 length of head, area between rugae microreticulated and moderately dull. Posterior part of head dorsum microreticulated, slightly dull, occiput smooth and shiny. Antennal scape with thin, longitudinal rugae. ***Mesosoma*.** Distinctly elongate. Promesonotum in dorsal view approximately 1.7 times as long as wide, pronotum strongly convex in profile. Anterolateral sides of pronotum convex, setose angulations or tubercles absent. Anterior part of mesonotum angulate or bituberculate, protruding distinctly above the level of posterior part of pronotum, thus promesonotal outline with distinct emargination in profile. Propodeum elongate, approximately 1.26 times as long as wide. Propodeal spines long, thin, at base only twice wider than at apex, acute apically, run only slightly upwards (Fig. 2). Dorsal part of pronotum with diffused microreticulation and only with median line smooth and shiny to mostly smooth and shiny, sides of pronotum with sparse, thin, mostly longitudinal rugae and diffused microreticulation between rugosities but appear shiny. Elevated part of mesonotum dorsally shiny with diffused microreticulation, laterally microreticulated with few rugae, posterior part of mesonotum rugose dorsally and granulate laterally (Fig. 2). Anterior surface of propodeum with short longitudinal rugae, laterally at least anteriorly smooth and shiny, posteriorly with few longitudinal rugae, dorsally with transverse or more or less longitudinal and around spiracles with irregular rugae. ***Petiole*.** Elongate with long peduncle, its anterior face deeply concave, node subangulate. Ventral margin of petiole in the middle straight, shallowly concave before apex, without spine or angulation. In dorsal view, petiole constricted at base then weakly divergent, almost parallel before petiolar node, then slightly globular. Base and ventral side distinctly microreticulated but without rugae, on sides and dorsally with diffused microreticulation to smooth and shiny, posterior faces with few rugae. ***Postpetiole*.** In lateral view rounded or slightly depressed at apex, in dorsal view approximately as long as wide with regularly rounded sides (Fig. 1). Base and ventral side distinctly microreticulated but without rugae, on sides and dorsally with diffused microreticulation to smooth and shiny, posterior faces with few rugae. ***Gaster*.** Shiny, with indistinct, diffused microreticulation, basal part of first tergite without or with very short longitudinal rugae. ***Setosity*.** Head in frontal view with short, light yellow, sparse setae. Entire dorsum of mesosoma and anterior margins of pronotum with sparse, short to moderately long, erect setae, the longest setae from shorter to approximately as long as propodeal spines. Petiolar node, postpetiole and gaster with short standing pilosity, the longest setae in large specimens shorter and in small specimens as long as propodeal spines.

**Figures 5, 6.**
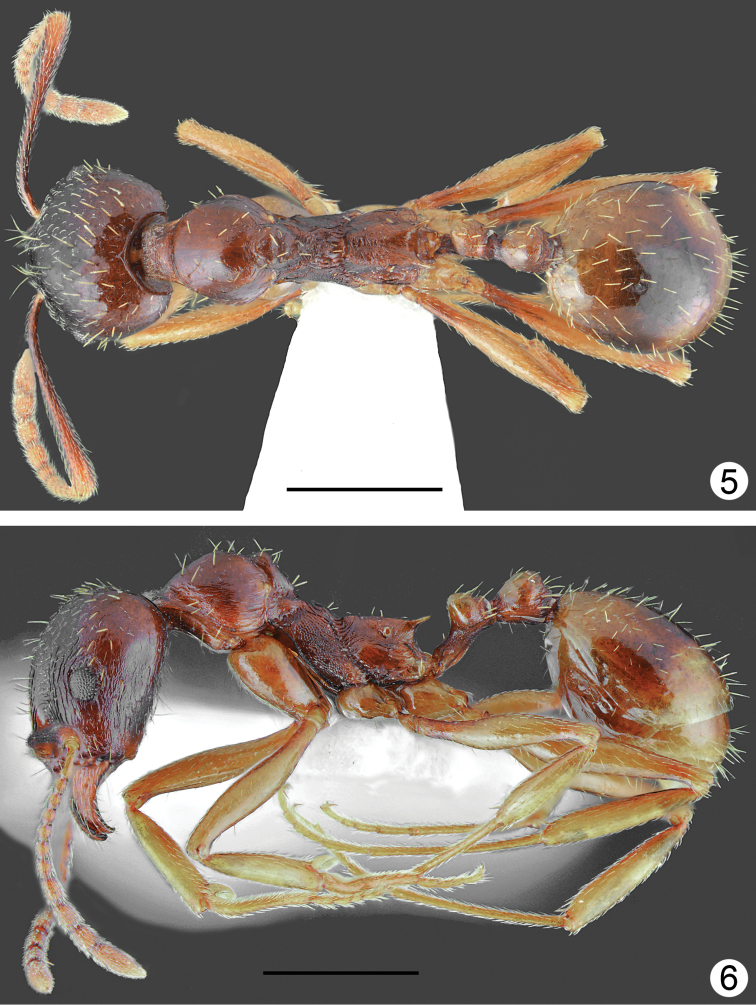
*Aphaenogasterillyrica*, paratype from Kamena, Bulgaria **5** dorsal **6** lateral. Scale bars: 1 mm.

**Figures 7, 8.**
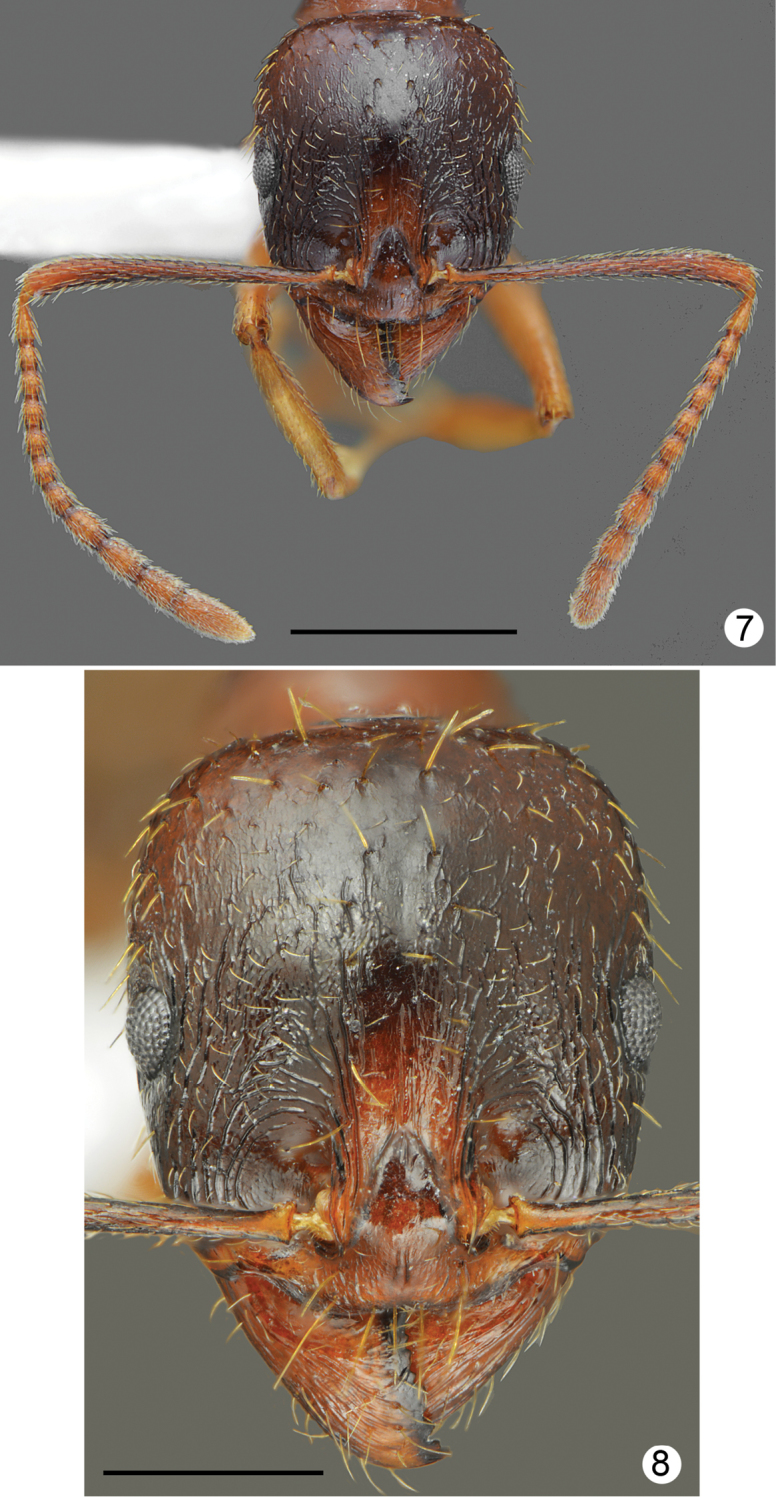
*Aphaenogasterillyrica*, paratype from Kamena, Bulgaria **7** head and antennae **8** head sculpture. Scale bars: 1 mm (**7**), 0.5 mm (**8**).

**Figures 9, 10.**
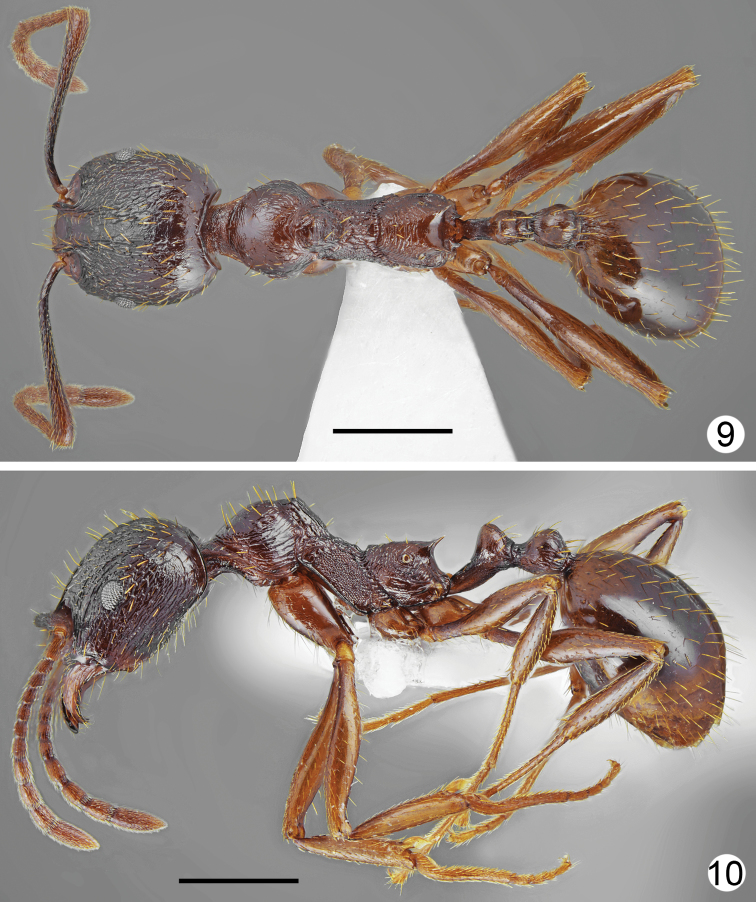
*Aphaenogastergraeca*, worker from Mt. Olympus **9** dorsal **10** lateral. Scale bars: 1 mm.

**Figures 11, 12.**
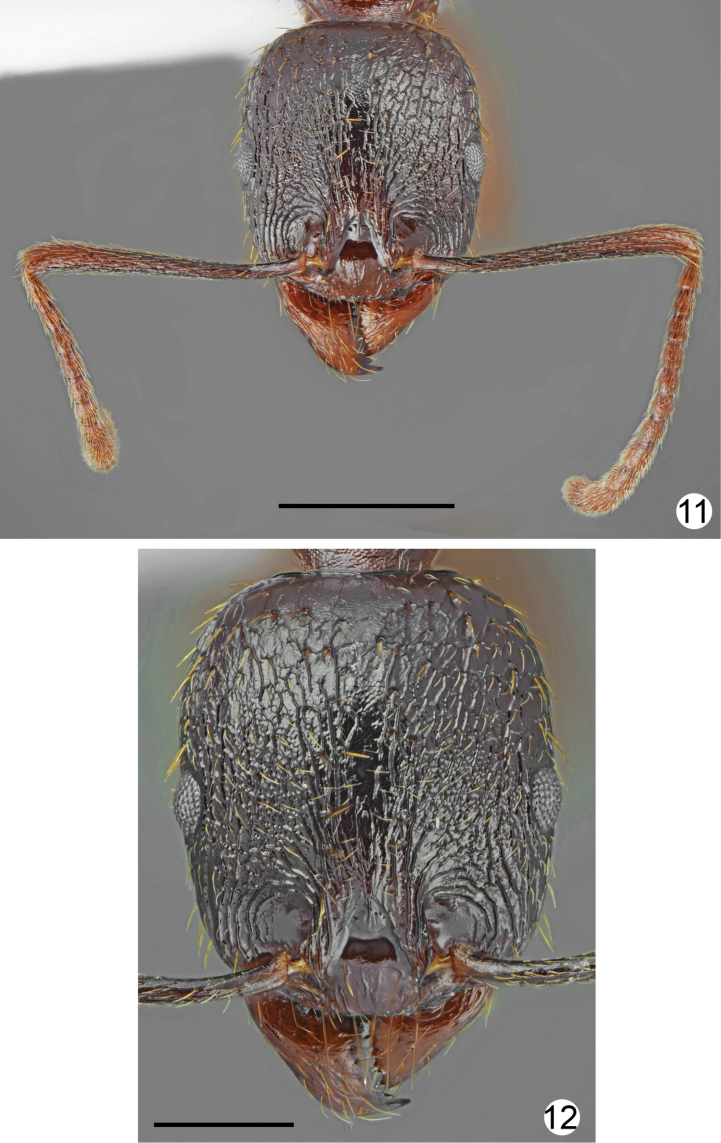
*Aphaenogastergraeca*, worker from Mt. Olympus **11** head and antennae **12** head sculpture. Scale bars: 1 mm (**11**), 0.5 mm (**12**).

**Figures 13, 14.**
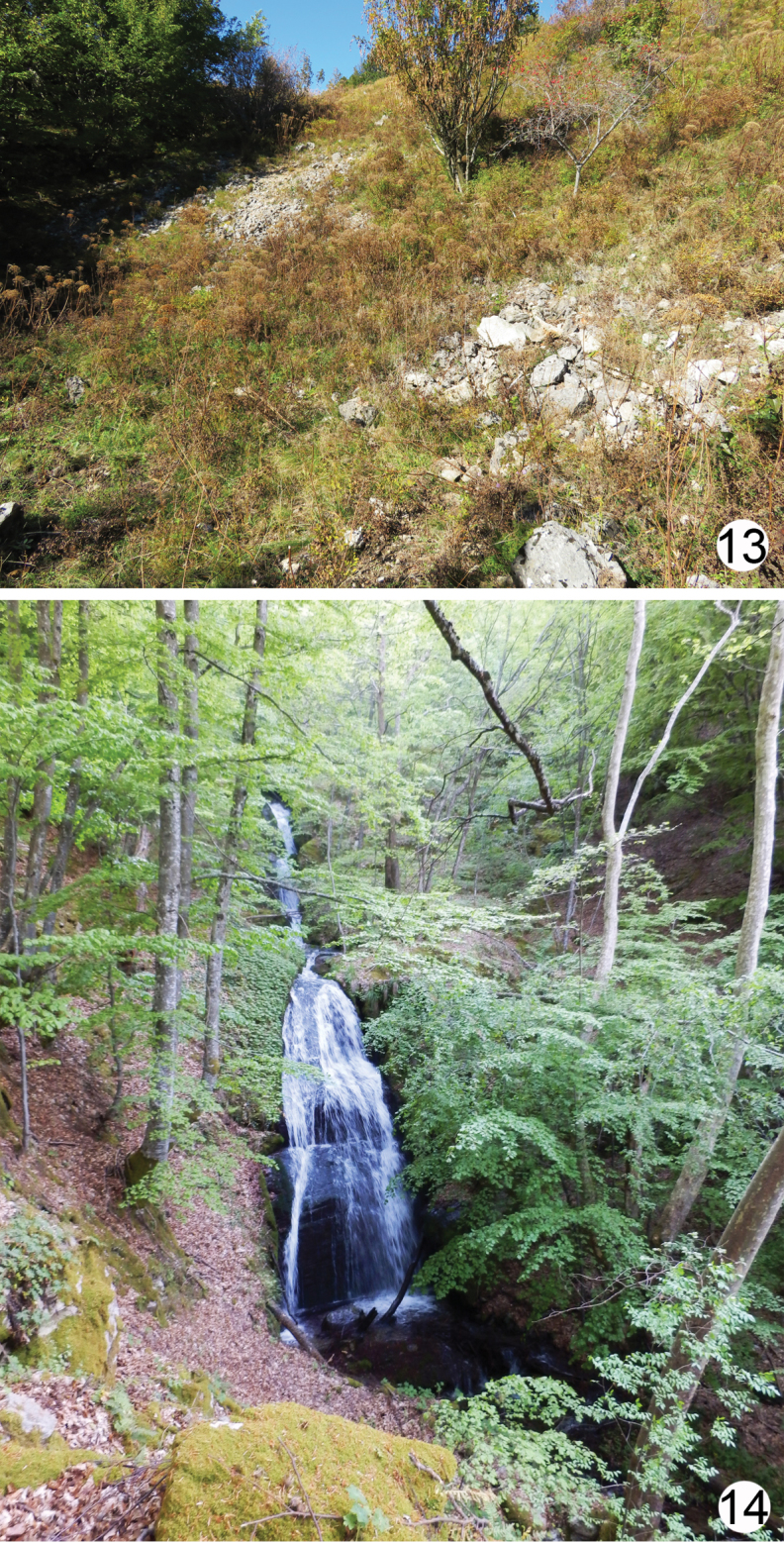
Habitat of *A.illyrica***13** locality Velika Milanja, Slovenia **14** locality Kamena, Bulgaria.

#### Gyne and male.

Unknown.

#### Range of the morphological variability.

Variability within the geographic populations of the new species *A.illyrica* is mostly in size of propodeal spines and distinctness of microreticulation of head occiput, dorsal part of pronotum, and mesopleuron. Variability between geographically distant populations is more distinct but features overlap. Specimens from Slovenia (terra typica) have the stoutest head (largest HI) while in samples from Croatia and Bulgaria head is less stout. Microreticulation on the dorsal surface of the head and on the dorsal part of the pronotum in specimens from Slovenia and Croatia is more distinct than in those from Bulgaria, and similarly the northern populations have more distinct longitudinal rugae on the sides of the pronotum. In contrast, reticulation of mesopleuron in Bulgarian samples is distinct on the whole surface while in some specimens from Croatia and Slovenia reticulation of the mesopleuron is partly diffused. In specimens of similar sizes, the propodeal spines are shorter and directed more or less upwards in northern populations, while in Bulgarian populations they are longer and almost in the prolongation of the upper edge of the propodeum, not or very slightly directed upwards.

#### Etymology.

Named after Illyria, a historical region in the western part of the Balkan Peninsula inhabited by the Illyrians and the ancient Roman Prefecture of Illyricum. All localities of *Aphaenogasterillyrica* are within the area of this region.

#### Distribution.

**Figure 15.**
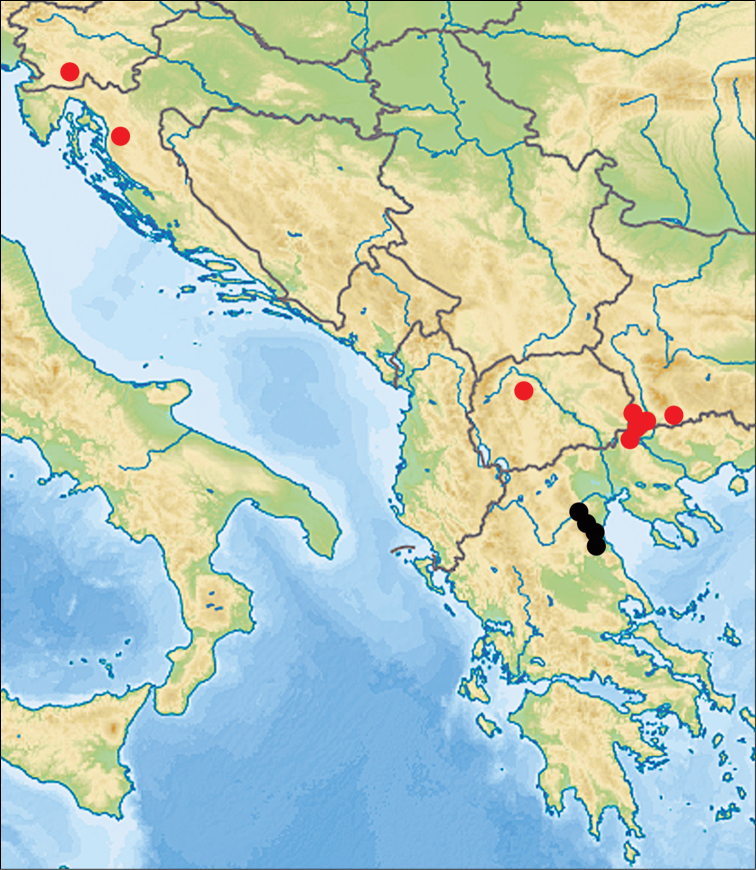
Distribution of *Aphaenogasterillyrica* (red circles) and *Aphaenogastergraeca* (black circles).

All known records of *Aphaenogasterillyrica* are restricted to the mountainous areas of the Balkan Peninsula, from the altitudes of 500 m to 1420 m a.s.l. Its range stretches from the Dinaric Alps in southern Slovenia and western Croatia to Osogovo-Belasica Massif in southwestern Bulgaria and the adjacent Kerkini Mts. in Greece and to Golešnica Mt. in North Macedonia. This distribution area is much larger compared to the area of the sister species *Aphaenogastergraeca*, whose distribution range is limited to the massif of Mount Olympus and adjacent mountain ranges (Fig. 15).

#### Biological notes.

Details on the new species habitat are available only from the Bulgarian and Slovenian records. In Bulgaria, *A.illyrica* was mostly collected in beech forests in wet sites, close to streams, on silicate (Belasitsa and Maleshevska Mts.) and limestone (Slavyanka Mt.) rocks. This differs quite dramatically from the Slovenian site, where the ants were found in a large karstic depression (karstic doline) situated in the sub-montane karst grassland, party covered with sparse trees and shrubs. This area is characterised by harsh winters and relatively wet summers. Due to the strong and almost permanent winds, the upper part of the soil is often dry. The specimens collected in 2003 were found in subterranean pitfall traps set in soil at the depth of 30–50 cm among the limestone rocks in the so-called Superficial Subterranean Habitat (SSH) or “Milieu Souterrain Superficiel” (MSS), as originally described (Juberthie et al. 1980; 1981). SSH is a hypogean environment, generally formed by the fragmentation of the bedrock and accumulation of debris, which contains a wide network of air-filled epikartsic spaces, small voids and fissures (Culver and Pipan 2009; Giachino and Vailati 2010) and represents a transition zone between surface soils and deeper subterranean habitats such as caves (Culver and Pipan 2009). A presence of a rare species *A.cardenai* Espadaler, 1981, was already reported from SSH in the Iberian Peninsula (Ortuño et al. 2014). In 2018, we found few scattered workers at the same site while digging in the stony ground to the depth of approximately 50 cm.

*Aphaenogasterillyrica* can be characterised as a ground-dwelling species. The records of *A.illyrica* well above 1000 m a.s.l. or those from beech forests at lower altitudes indicate that it tolerates lower temperatures, which is relatively rare in other species of the genus.

**Comments.** Recently published papers (Borowiec and Salata 2017, Alicata and Schifani 2019) indicate that the *A.subterranea* group is very diverse and comprise several undescribed taxa. The Balkans appears to be the most species-rich region and is in need of further investigation. Results presented in this publication are a preliminary attempt to systematise our knowledge about this group, and *Aphaenogasterillyrica* and *A.graeca* compose a distinct complex within the *A.subterranea* group. Therefore, we decided to describe the new species in a separate paper. Other undescribed forms, mentioned in the publication, will be a subject of further study. Because the *A.subterranea* group consists of mixture of species of uncertain taxonomic status and several undescribed morphotaxa, we can provide only a generic key to the *subterranea* group with features focused on the *graeca* complex.

##### A key to the *Aphaenogastergraeca* complex (within *A.subterranea* group)

**Table:** 

1	Metanotal groove absent or very shallow (Maghreb, Canary Islands, Siculo-Maltese archipelago, Southern Italy)	***A.crocea* group, *sensu*Alicata and Schifani (2019)**
–	Metanotal groove present, deep and narrow (Mediterranean Region)	**2**
2	Pronotum and mesonotum form regular convexity, mesonotum not raised above the surface of pronotum, propodeal spines short, not longer than half length of the first segment of antennal funiculus, mesosoma short	***A.subterranea* complex**
–	Mesonotum clearly raised above the surface of pronotum, propodeal spines long and thin, as long as or longer than 0.7 length of the first segment of funiculus, mesosoma elongated	**3**
3	Base of the first gaster tergite with distinct rugae, body brown to dark brown, head distinctly darker than mesosoma, head sculpture strong, posterior part of head dorsum with sculpture only slightly reduced but still distinct, lateral surface of propodeum, at least partly, with strong longitudinal rugae	*** A. graeca ***
–	Base of the first gaster tergite smooth, body uniformly yellowish-brown to rusty-brown, head sculpture weaker, posterior part of head dorsum with sculpture at least partly reduced, lateral surface of propodeum smooth or with few gentle longitudinal rugae	***A.illyrica* sp. nov.**

## Supplementary Material

XML Treatment for Aphaenogaster illyrica
